# An optimised clearing protocol for the quantitative assessment of sub-epidermal ovule tissues within whole cereal pistils

**DOI:** 10.1186/s13007-017-0217-z

**Published:** 2017-08-15

**Authors:** Laura G. Wilkinson, Matthew R. Tucker

**Affiliations:** 1 ARC Centre of Excellence in Plant Cell Walls and School of Agriculture, Food and Wine, University of Adelaide, Waite Campus, Urrbrae, SA Australia; 2 School of Agriculture, Food and Wine, University of Adelaide, Waite Campus, Urrbrae, SA Australia

**Keywords:** Microscopy, Hoyer’s solution, Barley, Ovule, Monocot, Development, Cereal, Clearing

## Abstract

**Background:**

Seed development in the angiosperms requires the production of a female gametophyte (embryo sac) within the ovule. Many aspects of female reproductive development in cereal crops are yet to be described, largely due to the technical difficulty in obtaining phenotypic information at the cellular or sub-cellular level. Hoyer’s solution is currently well established as a solution for clearing thin tissues samples, such as sections or whole tissues of bryophytes, mycorrhizal fungi, and small model organisms (e.g. *Arabidopsis thaliana*).

**Results:**

Here we report a Hoyer’s solution-based clearing method to facilitate clearing of the whole barley pistil, with high reproducibility. The clearing process takes 10 days from fixation to visualisation, whereupon tissue is sufficiently clear to obtain multiple phenotypic measurements from sub-epidermal tissues and cells within the ovule.

**Conclusion:**

Visualisation of cereal ovules that have not been dissected from the pistil allows an unprecedented capability to collect quantitative morphological information from the developing ovule, integument, nucellus and embryo sac. This will enable comparisons with genetic data to reveal the contribution of pre-fertilisation ovule tissues towards downstream seed development.

**Electronic supplementary material:**

The online version of this article (doi:10.1186/s13007-017-0217-z) contains supplementary material, which is available to authorized users.

## Background

Sustaining food production above the level of food demand is a growing global challenge. Estimates suggest that crop yields will need to increase by 25–75% to ensure sufficient food production for the world’s population in 2050 [1]. Cereal crop production is highly reliant upon development of flowers. In particular, the single ovule within each flower is essential, as it is the site of gametogenesis, fertilisation and downstream grain development. Environmental events such as drought, high temperatures and frost are known to disrupt flower and seed development, causing a reduction in both grain number and grain quality, thus compromising yield [2–4].

Our understanding of floral development and seed formation in flowering plants has been dramatically expanded by research in diverse model dicots, such as *Arabidopsis thaliana*, *Hieracium* sp., and *Torenia fournieri* [5–7]. The formation of ovule primordia, the differentiation of a megaspore mother cell from somatic precursors and the production and fertilisation of an embryo sac have been described in intimate molecular, genetic and morphological detail [8]. Research in rice, maize, wheat and barley has contributed significant molecular and genetic knowledge of monocot inflorescence and flower development [9–12]. Despite this, remarkably little is known about ovule development in these important cereal species, particularly in regards to how different tissues contribute to eventual seed size, composition and shape. Studies have shown that ovary size is an important component of floret and grain survival [13], but the contribution of constituent tissues remains unclear. Determining the role of these tissues for downstream seed development requires robust, high throughput methods for quantitative two and three-dimensional analysis of developing ovule tissues, such that phenotypic information can be extracted and assessed.

Observation of the internal morphology of cleared floral organs is a powerful tool that allows examination of phenotypic alterations in internal structures following genetic or environmental modification, without the need for thin-sectioning. Chemical treatment to clear small tissue samples is a well-established practice, with reagents ranging from the more traditional methyl salicylate, lactic acid and chloral hydrate based solutions [14–16] to recently developed methods such as ClearSee [17, 18] and PEA-CLARITY [19]. Despite this, observation of female reproductive tissues in cereal monocots remains technically challenging, contributing to a lack of specific genetic and mechanistic information about gametogenesis and ovule development. Two key technical challenges include the relatively large size of the pistils, which are sufficiently thick to remain opaque when treated using previously published clearing protocols designed for substantially smaller tissues (e.g. [20]), and the ease by which the physical structure of the ovule may be damaged during the process of dissection.

Here we report a robust method for clearing whole cereal pistils with Hoyer’s Solution [14], allowing visualisation of wheat and barley ovule ultrastructure in a manner that preserves the physical integrity of internal structures. Experimental variation of incubation time offers flexibility in sample preparation, yielding exceptionally clear tissue after a minimum of 10 days post tissue collection and up to a maximum of 16 weeks. The utility of the method was demonstrated by using optical sections through cleared pistils to measure the dimensions of component tissues, enabling phenotypic variation in ovule development to be captured within a panel of barley cultivars.

## Methods

### Reagents

- Chloral Hydrate C-IV (#15307, Sigma-Aldrich, Australia)
- Ethanol (#EA043-2.5L, Chem-Supply, Australia)
- Formaldehyde (#809, Ajax Finechem, Australia)
- Glacial Acetic Acid (#2335, Ajax Finechem, Australia)
- Glycerol (#242, Ajax Finechem, Australia)

### Solutions

- *FAA fixative* [21] 50% Ethanol (v/v), 10% Formaldehyde (37% solution, also called formalin), 5% glacial acetic acid (v/v), and 35% sterile water (v/v).
- *Ethanol Series* 100% analytical grade EtOH diluted in water to a concentration of 70, 80 and 90%, and 100% EtOH filtered through a molecular sieve.
- *Chloral hydrate solution* 250 g chloral hydrate dissolved in 100 mL sterile water
- *Hoyer’s Solution* [14] A 3.0:0.8:0.2 mixture of chloral hydrate:water:glycerol.

### Equipment

- Greenhouse facility
- Standard laboratory 4 °C refrigerator
- Fume cupboard
- Compound microscope with differential contrast (DIC) and Nomarski filter for a ×10, ×20 and/or ×40 objective
- Computer and free ZEN 2011 Blue (Zeiss) LE software
- Ventilated microscopy slide box
- Small exhaust fan
- Glass pipettes
- Fine point tweezers (Dumont #5, Emgrid, Australia)
- Liquid scintillation vials (#Z190535, SigmaAldrich, Australia)
- Polysine Slides (#P4981, ThermoFisher Scientific, Australia)
- 22 × 40 mm Cover slips (#G422, ProSciTech, Australia)
- Microflex 93-260 chemical resistant gloves (Ansell, Australia)

### Plant growth and staging

**Fig. 1:**
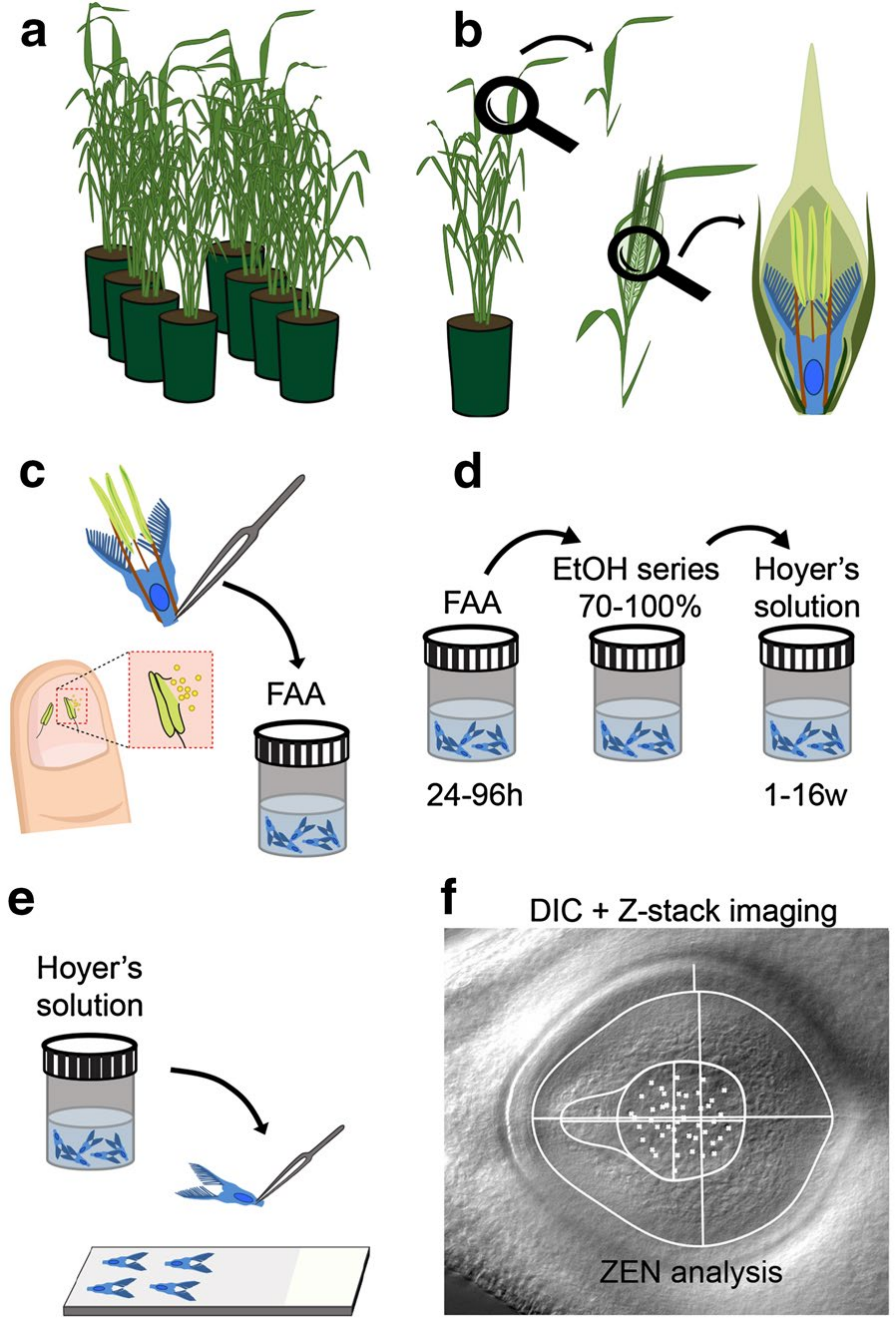
Schematic representation of the steps involved in visualising the internal structures of ovules within cleared cereal pistils. **a** Plants were examined to identify tillers containing developing spikes. **b**, **c** Individual florets were removed from spikes to identify those at anthesis stage, achieved via observation of pollen on fingernails after anther squashing. **c**, **d** Whole pistils and anthers were gently removed and placed in fixative, followed by dehydration in an ethanol series and clearing in Hoyer’s solution. **e** Pistils were transferred to glass slides and covered with glass coverslips. **f** Samples were examined using DIC microscopy and Zeiss ZEN software

Barley plants were grown in greenhouse facilities at The Plant Accelerator (Adelaide, Australia), under 22 °C (day) and 17 °C (night) temperatures without addition of supplemental light (Fig. 1a). Florets were identified to be at anthesis by removing them from spikes (Fig. 1b), gently reaching inside the palea and lemma with tweezers then assessing the colour of the anthers and how readily pollen was released upon gentle squashing. At anthesis, the anthers are a rich yellow colour and have yet to shed pollen, but readily release pollen with minimal application of pressure when pressed against a thumbnail (Fig. 1c). Any florets that contained green or green/yellow anthers, or anthers that had already shed pollen, were discarded.

### Sample collection and fixation (timing: 10 min per tiller + overnight fixation)

Whole pistils were removed from anthesis barley flowers by reaching inside the flower with fine tweezers and pinching the base of the pistil as low as possible (Fig. 1c). Care was taken to avoid tearing the base of the pistil where the ovule is located. Lodicules were gently removed from the outside of the pistil before placing it in a flat bottomed glass scintillation vial containing 2 mL of ice cold FAA fixative.

### Sample dehydration (timing: 4 h + overnight dehydration + 4 to 120 days incubation)

Within 1 week of fixation barley pistils were dehydrated through an ethanol series and placed into Hoyer’s solution (Fig. 1d), using fine-tipped glass pipettes for each fluid exchange to minimise the possibility of damage to tissue samples. The EtOH series comprised of 3 × 20 min washes at 70, 80, 90 and 100% EtOH at room temperature. Samples were left in the final 100% EtOH wash overnight before transfer into 4 mL Hoyer’s Solution. Samples must remain immersed in Hoyer’s solution at room temperature for a minimum of 4 days.

#### *Long protocol*

Samples may remain gently infiltrating in Hoyer’s solution for up to 16 weeks. Incubation for 4 weeks preserves tissue quality ideally for imaging of embryo sac features. Vials must be tightly sealed if samples are to be stored for longer than 2 weeks.

### Sample mounting (timing: 15 min per slide + 2 to 4 days incubation)

Pistil tissues were manipulated with fine point tweezers and only held by the stigma in order to avoid crushing the ovary wall, ovule or surrounding tissue. Pistils were placed on flat Poly-Lys coated glass microscopy slides with either the dorsal or ventral side down so that both stigma of each pistil lay “flat”, rather than one stigma pointing up into the air (Fig. 1e). On each slide, pistils were placed equidistantly in a symmetrical arrangement and gently covered with a 22 × 40 mm coverslip. This arrangement allows the pistils to lie flat, ensures that variation in the relative viewing angle of the ovule is limited, and preserves the structural integrity of the ovule by preventing any damage to the tissue. Following sample arrangement and application of the cover slip, Hoyer’s Solution was pipetted underneath the cover slip onto the slide until all air was evacuated. Slides were then placed flat into a slide storage box that allowed limited ventilation and left in a fume cupboard for 4 days. Samples stored in a well ventilated location are cleared in 24–48 h depending upon the degree of ventilation. Conversely, samples stored after mounting with insufficient or no ventilation required up to 14 days to clear sufficiently to allow visualisation. Therefore, the degree of ventilation can be used to tailor the method to suit the user’s time constraints.

#### *Long protocol*

Samples incubated in 4 mL Hoyer’s Solution for longer than 2 weeks typically require less than 4 days to clear completely once mounted on the microscopy slide. For example, tissue stored in Hoyer’s Solution for 8–16 weeks generally does not require a period of ventilated storage longer than 12 h, and in some cases may be visualised immediately after mounting on slides.

### Imaging (timing: 2 min per piece of tissue)

Pistils were imaged using differential contrast microscopy (DIC) at ×10 magnification with a Zeiss AxioImager M2 equipped with a Nomarski filter. For comprehensive data collection, optical slices spanning from the dorsal to ventral integument were taken as a z-stack image, using Zeiss ZEN 2011 (Blue) software.

### Image analysis (timing: 10 to 15 min per image)

Data were analysed using the Zeiss ZEN 2011 (Blue) software package. Diverse measurements were taken including the 2-dimensional area (μm^2^) of each ovule tissue of interest, using the “contour (spline)” graphics tool to encircle the tissue, as well as the longitudinal and transverse dimensions (μm) of the same tissues, using the “line” graphics tool, and the antipodal nuclei were counted using the “event marker” graphics tool (Fig. 4c). Measurements were taken by following tissue boundaries for each given trait throughout optical sections and placing contour markers at the widest point. Two-dimensional ovule area was measured at the boundary between integument and nucellus. Embryo sac area was measured by tracing the outline of the structure from the micropyle to the chalazal region. The residual somatic cell (nucellus) area was measured by subtracting the embryo sac area from the whole ovule area.

## Results

### Protocol timing optimisation

**Fig. 2:**
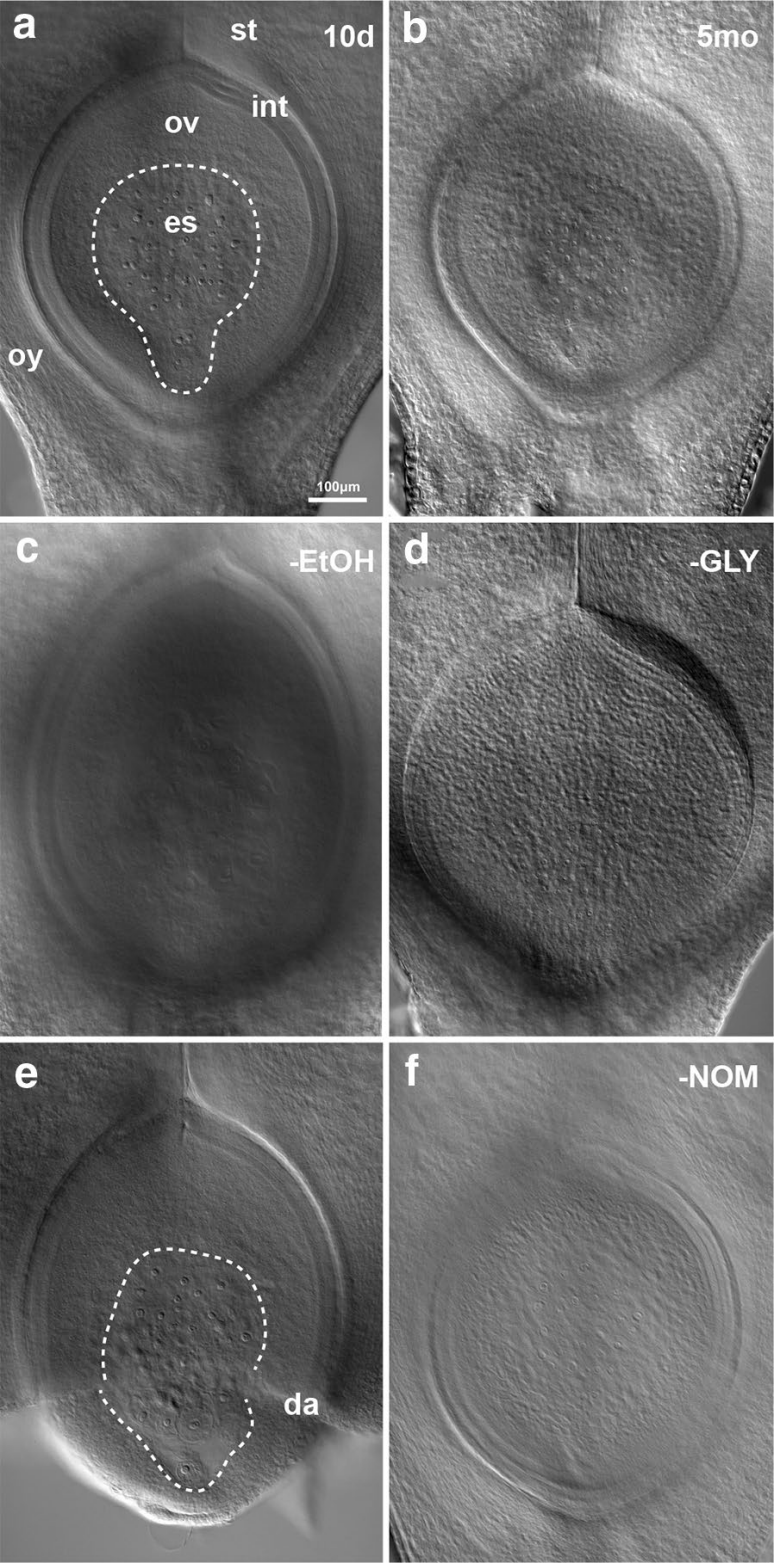
Barley ovules imaged at ×10 showing the outcomes of variations to the clearing protocol. Images presented as composites, generated by merging optical sections. **a** A 10-day (10d) method incorporating ethanol dehydration prior to a 4-day infiltration with Hoyer’s solution, then a 4-day rest after mounting on microscopy slides produced the greatest clarity of results within a reasonably short time frame. es = embryo sac, ov = ovule, oy = ovary wall, st = style, int = integuments. **b** Samples gently infiltrated with Hoyer’s solution for over 5 months (5mo) deteriorated, resulting in unacceptably murky images. **c** Omitting ethanol (-EtOH) dehydration prior to incubation in Hoyer’s solution results in the tissue becoming grainy and unacceptably murky. **d** Incubation of the sample in chloral hydrate without glycerol (-GLY) after fixation and dehydration results in the tissue becoming unacceptably murky. **e** Rough sample collection and careless handling of the tissues results in damaged ovaries, which may disrupt the internal morphology of the ovule. da = damaged region. **f** Samples cannot be imaged without a Nomarski filter (-NOM)

Clearing was most successful when fixative was removed through an ethanol dehydration series prior to a 4-day infiltration step in Hoyer’s solution, followed by a 4-day rest after mounting on microscopy slides (Fig. 2a). Equally clear images were obtained from samples that were dehydrated, left to gently infiltrate in Hoyer’s solution for 4 weeks, then imaged directly after mounting on microscopy slides (Additional file 1: Fig S1A). The maximum period of incubation that achieved acceptable clearing was approximately 16 weeks (Additional file 1: Fig S1B). Deterioration of cellular morphology was seen when samples were left in scintillation vials to gently infiltrate with Hoyer’s solution for longer than 5 months (Fig. 2b), or when samples were mounted on microscopy slides and stored in a well ventilated area for multiple days, or were imaged after 10 days in a semi-ventilated storage box (Additional file 1: Fig S1C). Evaporation of the Hoyer’s solution was also a factor that prevented acquisition of acceptable images if the samples were over-ventilated.

### Protocol reagent optimisation

Clearing was not successful when ethanol dehydration was omitted and fixed samples were placed directly in Hoyer’s solution (Fig. 2c). Similarly, it was found that use of pure chloral hydrate solution rather than Hoyer’s solution yields unacceptably murky images (Fig. 2d), a factor of both the harsher degradation process when chloral hydrate is used in isolation and the lack of glycerol lowering the refractive index of the mounting fluid. Rough sample collection or handling of tissue throughout the dehydration process often resulted in structural disruption of the sample (Fig. 2e; Additional file 1: Fig. S1D). In addition, a Nomarski filter is essential for image acquisition (Fig. 2f).

### Optimised method results

**Fig. 3:**
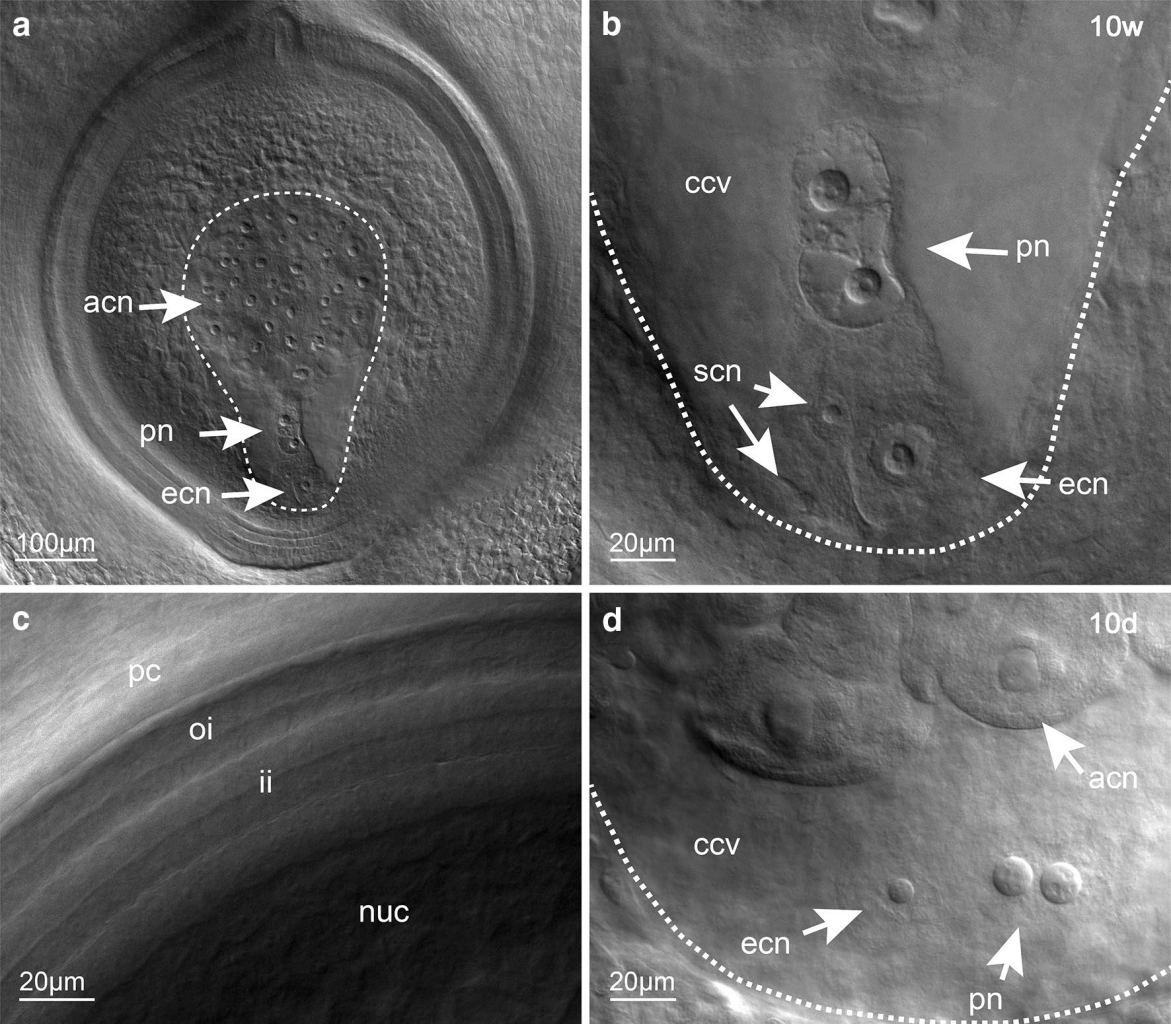
Structural details of the ovule are visible in cleared barley pistils (*H. vulgare* cv. Host). Images presented as composites, generated by merging optical sections. **a** Mature barley ovule imaged at ×10 magnification without dissection from the pistil. **b**–**d** Cellular resolution may be achieved with a ×40 objective, allowing clear visualisation of the egg cell nucleus, synergid nuclei, polar nuclei and antipodal cell nuclei using the “long method” i.e. incubation for at least 10 weeks (10w) in Hoyer’s solution (**b**). The integument layers are also visible (**c**). Similar cellular resolution may also be achieved in samples processed with a 10-day (10d) “short” method (**d**). acn = antipodal cell nuclei, ccv = central cell vacuole, ecn = egg cell nucleus, ii = inner integument, nuc = nucellus, oi = outer integument, pn = polar nuclei, pc = pericarp, scn = synergid cell nuclei. The embryo sac is indicated by a *dashed white line*

**Fig. 4:**
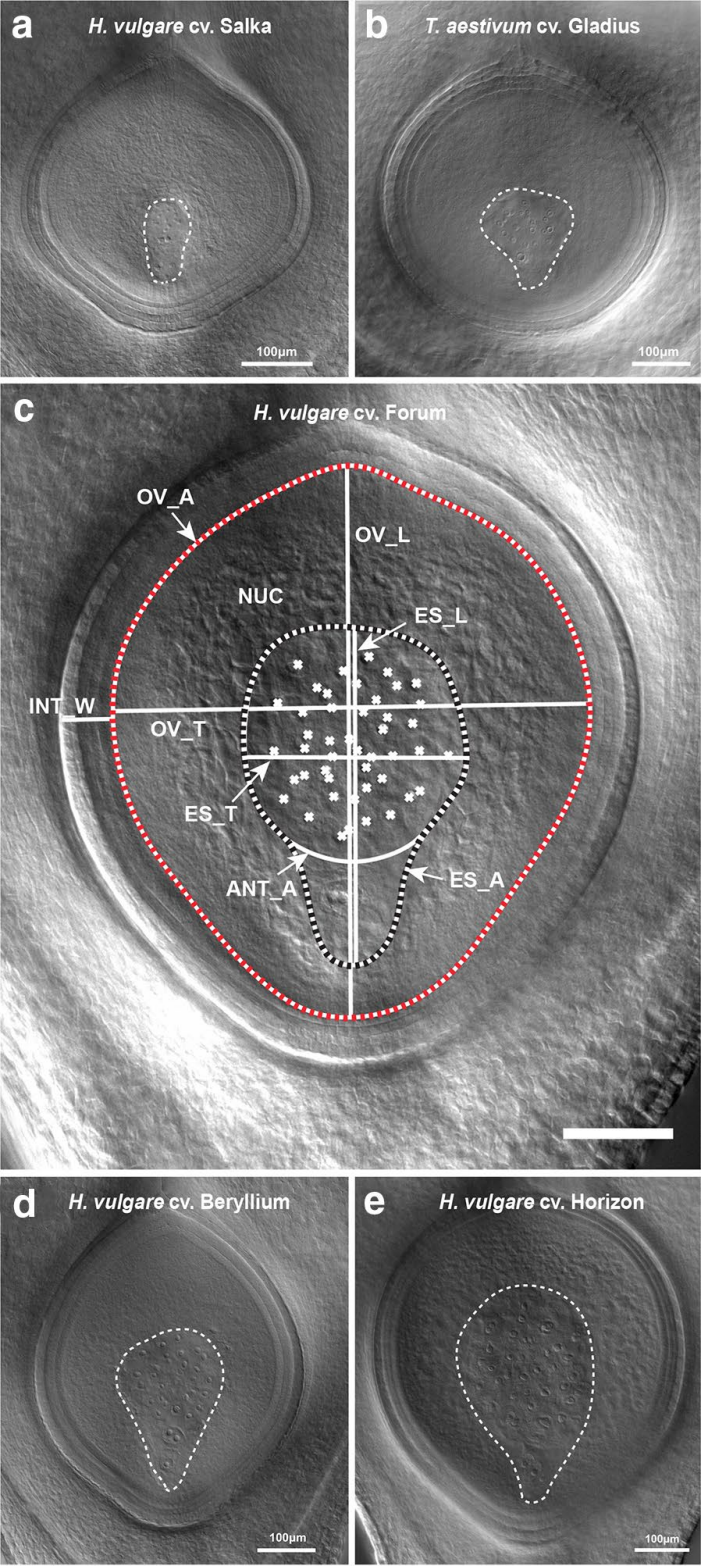
Ovule morphology after clearing. Ovule images are presented as composites, generated by merging optical sections. The method worked equally well for barley (**a**) and wheat (**b**) pistils at various stages. Pre-anthesis ovules are shown. **c** Measurement of traits at anthesis, including ovule area (OV_A), embryo sac area (ES_A), ovule and embryo sac transverse and longitudinal dimensions (OV_L, OV_T, ES_L, ES_T), integument width (INT_W), antipodal number (*marked with crosses*) and antipodal cluster area (ANT_A), using Zeiss ZEN software. The ovule area is indicated with a *dashed red* and *white line*, while the embryo sac area is indicated by a *dashed black* and *white line*. NUC = nucellus. Small (**d**) and large (**e**) mature barley ovules after clearing imaged at ×10 magnification without dissection from the pistil. *Bar* 100 μm

Cleared pistils offer an excellent opportunity to visualise internal components of the ovule in their native spatial arrangement using a DIC microscope with a Nomarski filter (Figs. 3, 4). Imaging the entire ovule within the ovary is easily possible at ×10 magnification, and is particularly powerful when captured in a series of optical sections, allowing construction of composite images and videos that represent all internal features of the ovule’s cellular arrangement (Fig. 3a; Additional file 2: Fig. S2) and measurement of some three-dimensional features such as embryo sac depth. At ×40 magnification, intimate cellular details of the embryo sac and other ovule components could be obtained (Fig. 3b, c), such as clear, prominent nuclei in the egg cell, central cell and antipodal cells. The quality of tissue resolution was similar in pistils that were infiltrated in Hoyer’s solution for 10 days (Fig. 3d) and 10 weeks (Fig. 3b).

### Sup-epidermal details of ovule development differ between cultivars

**Fig. 5:**
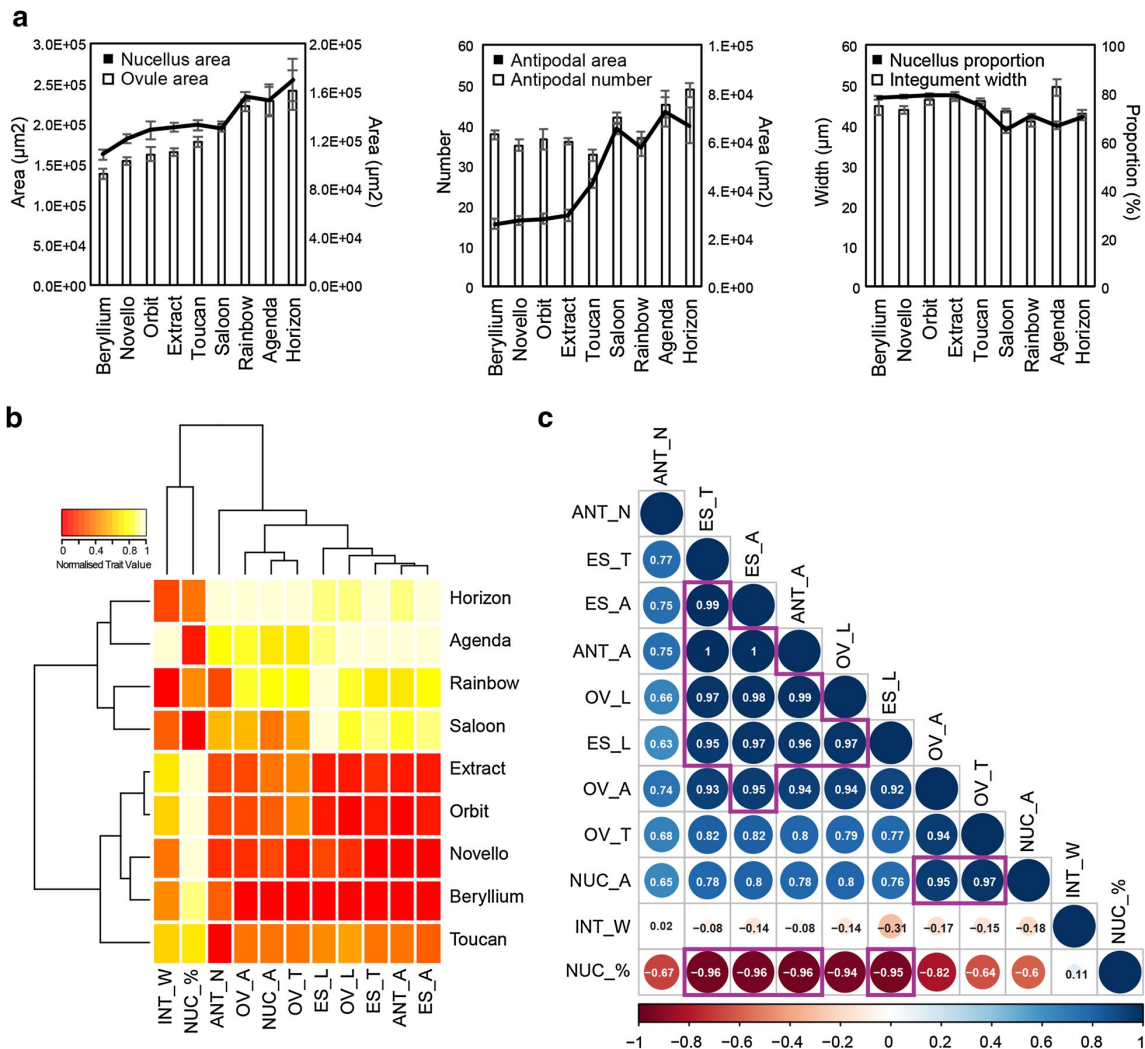
Analysis of barley ovule traits by pistil clearing. **a** Ovule phenotypes were examined in nine cultivars of 2-row spring barley. Traits such as ovule area, nucellus area, antipodal number, antipodal area, nucellus proportion and integument width (see Fig. 4) were compared between the cultivars. *Error bars* show standard error. **b** Heat map showing the normalised trait values (between 0 and 1) for 11 ovule traits in the 9 examined cultivars. Cultivars and traits were clustered via hierarchical clustering. **c** Correlation analysis of 11 different ovule traits. The *size* and *colour* of the *circles* indicates the degree of trait correlation, which is also indicated via a numerical value. R-squared values greater than 0.95 are indicated via *purple boxes*. INT_W = integument width, NUC_% = nucellus proportion, ANT_N = antipodal number, OV_A = ovule area, NUC_A = nucellus area, OV_T = ovule transverse width, ES_L = embryo sac longitudinal height, OV_L = ovule longitudinal height, ES_T = embryo sac transverse width, ANT_A = entire antipodal area, ES_A = embryo sac area

A fundamental understanding of reproductive organ development in cereals ultimately aims to support breeding programs in generating high-yielding, high-quality cultivars. To demonstrate the utility of this clearing technique, we examined pistils from barley and wheat (Fig. 4a, b). In both species, sub-epidermal details of ovule tissues, including the embryo sac, egg cell, central cell, antipodals, integument and nucellus could be discerned and measured (Fig. 4c). To determine if intraspecific differences in ovule development could be identified, we examined a selection of 2-row spring barley cultivars. Quantification of morphological features such as tissue area, thickness and cell number in nine cultivars revealed natural variation in most traits (Table 1; Figs. 4d, e, 5). For example, ovule area in *H. vulgare* cv Horizon was almost twofold larger than *H. vulgare* cv Beryllium (Figs. 4d, e, 5a), antipodal number was lowest in *H. vulgare* cv. Toucan (~32 ± 5) compared to *H. vulgare* cv. Horizon (~49 ± 4) and integument width was thickest in *H. vulgare* cv Agenda (~50 ± 4 μm) and thinnest in *H. vulgare* cv Rainbow (~41 ± 3 μm). Correlation analysis indicated that multiple traits showed strong positive correlations, such as ovule area, embryo sac area and ovule height (Fig. 5b, c), suggesting that these features are intimately related. However, other traits showed weak or no correlations with other ovule features, including integument width, antipodal number, nucellus area and ovule transverse width (Fig. 5b, c).

**Table 1:** Phenotypic measurements of ovule tissues from nine *H. vulgare* cultivars

Cultivar	n	Ovule			Embryo sac			Integument	Nucellus		Antipodal	
		Area (μm^2^)	Trans (μm)	Long (μm)	Area (μm^2^)	Trans (μm)	Long (μm)	Width (μm)	Area (μm^2^)	%	#	Area (μm^2^)
Beryllium	6	138,197.8	396.9	484.3	30,462.7	174.6	270.1	44.9	107,735.1	78.1	37.7	25,829.6
STDEV		15,888.7	28.7	21.7	5597.7	26.0	25.5	5.7	11,329.2	2.2	2.7	5562.9
Novello	12	154,239.1	413.1	505.6	33,330.3	171.7	298.0	43.9	120,908.9	78.5	35.0	27,401.0
STDEV		16,522.7	19.1	42.8	6097.1	16.7	37.4	3.2	12,418.2	2.5	4.7	6447.3
Orbit	6	161,883.0	451.7	487.3	33,816.5	182.2	281.0	46.4	128,066.6	78.9	36.5	28,026.6
STDEV		20,556.0	32.4	20.9	4352.4	17.3	10.6	3.0	18,494.2	2.6	6.6	4934.9
Extract	11	164,652.1	448.3	497.6	34,479.2	188.9	284.0	46.7	130,172.9	79.1	36.0	29,457.8
STDEV		14,406.3	22.8	21.5	7094.9	25.8	27.4	2.7	11,537.1	3.5	2.9	7992.9
Toucan	11	177,561.6	444.7	554.2	45,330.7	210.7	320.3	46.1	132,230.9	74.7	32.6	42,617.2
STDEV		21,683.1	25.0	44.8	9374.7	22.8	46.4	2.1	14,255.4	3.3	4.7	5469.6
Saloon	13	199,385.6	456.7	598.7	70,066.1	266.1	382.3	43.6	129,319.5	64.9	41.8	65,125.7
STDEV		11,367.7	19.1	25.6	9071.9	21.7	25.0	1.7	10,869.6	3.9	4.8	8283.8
Rainbow	6	221,620.1	488.6	598.6	65,471.9	242.4	375.2	41.0	156,148.2	70.6	36.8	57,424.8
STDEV		14,500.2	20.3	19.8	8707.8	16.8	23.0	3.0	8030.2	2.5	3.5	7982.3
Agenda	6	227,499.2	483.4	625.2	75,121.6	272.9	367.9	49.5	152,377.7	66.7	45.2	72,000.8
STDEV		42,467.0	42.0	60.6	13,856.8	28.5	53.2	4.9	32,315.5	4.3	8.5	14,718.3
Horizon	6	241,310.7	519.2	607.2	72,158.9	268.8	371.9	43.0	169,151.8	70.0	48.8	66,487.6
STDEV		48,703.9	48.0	59.0	14,691.2	29.0	48.1	1.5	35,130.8	2.2	3.6	14,851.0

*Trans* transverse width, *Long* longitudinal height

## Discussion

In this study a method for clearing tissue using Hoyer’s solution has been designed to suit cereal pistils such that internal structures of the ovule may be imaged with a high degree of clarity. Chloral hydrate-based clearing solutions have been successfully used in a wide range of biological fields [14, 22, 23], permitting a great deal of fundamental morphological and phenotypic information to be gathered. However, in our hands, previously reported protocols incorporating chloral hydrate that work well in Arabidopsis (e.g. [20, 24]) did not result in sufficient clearing of barley pistils to enable quantitative measurement of individual ovule tissues. Moreover, alternative methods that incorporate methyl salicylate [16, 25–27], lactic acid [28], sodium hypochlorite [29] or sodium hydroxide [30], lack the convenience and/or efficiency of our established Arabidopsis chloral hydrate-based method [20]. Other recently reported clearing reagents such as ClearSEE [17], PEA-CLARITY [19] and FocusClear [31] are designed to clear tissue while preserving fluorescent labelling, but are either too expensive for high-throughput analysis or provide insufficient cellular resolution without additional staining.

Although the chloral hydrate-based method we describe is not compatible with visualisation of fluorescently-tagged proteins, it can be applied to diverse cereals, allows customisable incubation times, requires minimal tissue handling, and consistently provided excellent clearing and an ability to detect quantitative differences in tissue development in unstained cereal ovary samples. The Zeiss ZEN software used for image analysis is freely available for download and easy to use, while the FIJI software suite was used to extract similar results [32]. In our pilot study of barley ovules at anthesis, 75 pistils were examined from 9 cultivars. The method was not specifically tested on a microscope containing a motorised 8-slide mounting frame or image stitching software, but such an approach would almost certainly be compatible, suggesting that image acquisition might be automated in future to allow for high-throughput data collection. Whether the scale of analysis required for germplasm screens in breeding populations can be achieved is currently unclear. However, the method is compatible with pre-breeding efforts to dissect pre-fertilisation traits that contribute to downstream seed development and morphology. Furthermore, we anticipate that the method will be particularly useful for the rapid characterisation of mutant phenotypes and transgenic plants that effect ovule development in barley and wheat.

## Conclusions

A clearing technique typically used in the analysis of tissues from dicot model organisms was successfully adapted to clear the much larger cereal pistil. This paves the way for further interrogation of sup-epidermal features of ovule development in barley and other cereal crop species. The application of this method to a large panel of genetically distinct or genetically modified cereal varieties may assist the identification of novel genes controlling ovule phenotypes as well as components of seed yield and quality.

## Additional files

**Additional file 1: Fig.** **S1.** Barley ovules imaged at ×10 showing the outcomes of variations to the clearing protocol. Images presented as composites, generated by merging optical sections. **A** Ethanol dehydration prior to a 4-week (4w) gentle infiltration with Hoyer’s solution, then imaging samples directly after mounting on microscopy slides produced high clarity results in a longer time frame. **B** Samples gently infiltrated with Hoyer’s solution for 16 weeks (16w) then immediately imaged produced high-quality results. **C** Samples left mounted on microscope slides in a well ventilated storage box or for too long were not able to be imaged properly due to evaporation (+Evap) of the Hoyer’s solution, causing uneven illumination of the sample and in some cases accelerated degradation of the tissue, resulting in an unacceptably grainy image. **D** Rough sample collection and careless handling of the tissues results in damaged ovaries, which may disrupt the internal morphology of the ovule. The embryo sac is indicated by a dashed white line.

**Additional file 2: Fig.** **S2.** Sequential 2.4 μm optical slices (n = 50) of a cleared *H. vulgare* cv. Gant ovule at anthesis were combined to generate a movie file.
