# Supplementary figures and images for: An optimised clearing protocol for the quantitative assessment of sub-epidermal ovule tissues within whole cereal pistils
Source: Plant Methods. 2017 Aug 15;13:67. doi: 10.1186/s13007-017-0217-z (PMC5558753; doi:10.1186/s13007-017-0217-z)

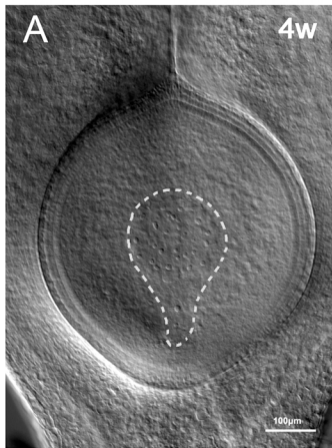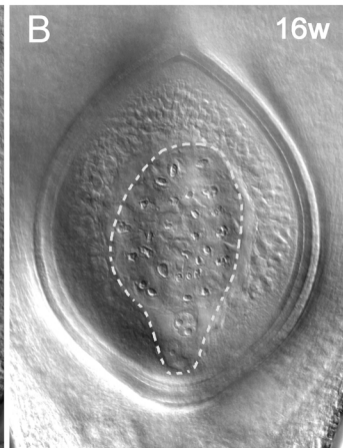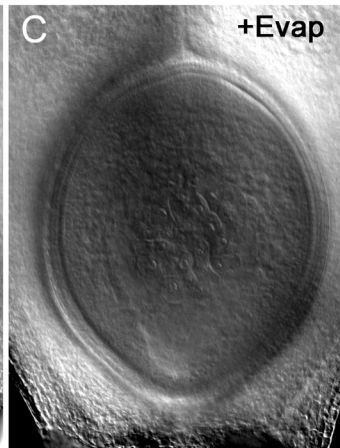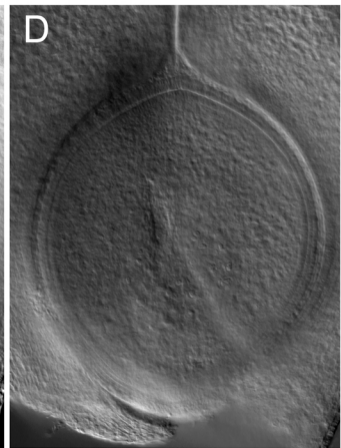

Supplement: Supplementary file 1 — Additional file 1: Fig. S1. Barley ovules imaged at ×10 showing the outcomes of variations to the clearing protocol. Images presented as composites, generated by merging optical sections. A Ethanol dehydration prior to a 4-week (4w) gentle infiltration with Hoyer’s solution, then imaging samples directly after mounting on microscopy slides produced high clarity results in a longer time frame. B Samples gently infiltrated with Hoyer’s solution for 16 weeks (16w) then immediately imaged produced high-quality results. C Samples left mounted on microscope slides in a well ventilated storage box or for too long were not able to be imaged properly due to evaporation (+Evap) of the Hoyer’s solution, causing uneven illumination of the sample and in some cases accelerated degradation of the tissue, resulting in an unacceptably grainy image. D Rough sample collection and careless handling of the tissues results in damaged ovaries, which may disrupt the internal morphology of the ovule. The embryo sac is indicated by a dashed white line. [file 13007_2017_217_MOESM1_ESM.pdf]
